# Breaking the Silence: Barriers to Fertility Preservation Among Young Breast Cancer Patients in Ghana – a multicenter study

**DOI:** 10.21203/rs.3.rs-6649154/v1

**Published:** 2025-11-19

**Authors:** Promise E. Sefogah, Alim Swarray-Deen, Josephine Nsaful, Dzifa A. Attah, Cheryl A. Moyer, Samuel A. Oppong

**Affiliations:** University of Ghana Medical School; University of Ghana Medical School; University of Ghana Medical School; University of Ghana Medical School; University of Michigan Medical School; University of Ghana Medical School

**Keywords:** fertility, preservation, breast, cancer, treatment, barriers, women

## Abstract

**Background:**

Breast cancer is the most common cancer among women in Ghana and a leading cause of cancer-related mortality. While incidence increases with age, many cases occur in women of childbearing age, often a decade earlier than in Caucasian populations. Although advances in treatment have improved survival, chemotherapy and radiation therapy carry a risk of infertility. Despite global recommendations for early fertility preservation (FP) counselling, limited data exist from Ghana.

**Methods:**

A cross-sectional, multicentre study using a quantitative survey was conducted at Korle Bu Teaching Hospital and the National Radiotherapy Oncology Centre. Eligible participants were reproductive-aged women with breast cancer. Exclusions included women who had completed childbearing or were not intending to have biological children. Data on sociodemographic, cancer treatment, FP awareness, and barriers were collected using structured questionnaires and analysed in SPSS v22.

**Results:**

Among 300 participants (mean age 44.5 years), FP awareness was 39.3%. Major barriers included cost (73.5%, p < 0.001), fear (50%), and partner objection (14.3%). Younger age, low parity, and single status were significantly associated with FP preference.

**Conclusion:**

Cost, limited awareness, and sociocultural factors impede FP uptake. Integrating fertility counselling into cancer care could improve outcomes for young breast cancer patients.

## INTRODUCTION

Globally, breast cancer is the most diagnosed cancer and the leading cause of cancer death among women, especially in low-resource countries. (1) While breast cancer is the second most prevalent cause of cancer death among women in high-income countries, in Ghana, breast cancer is the most common cancer among women and accounts for the highest burden of cancer mortality in women. (2)

Although the incidence of breast cancer increases with age, it has become the most diagnosed malignancy in women of childbearing age, as many women now delay childbearing. The peak age for breast cancer in Ghana has been reported to be ten years below that for the Caucasian population. (3) A study by Ghartey FN, et al, reported the average age for breast cancer diagnosis in Ghana as 38 years, with 30% of affected women being diagnosed below 35 years. (3) Notably, these women present with advance disease, often requiring gonadotoxic chemotherapy that increases the risk of subsequent fertility impairment.

With increasing survival rates from breast cancer due to advances in medical therapeutic interventions, there is an associated increase in the number of young women breast cancer survivors with fertility wishes and needs. (4)

Cancer treatment in general may influence female reproduction through various mechanisms. Notably, at the level of the gonads, chemotherapy, whole-body or pelvic only radiation may result in radiation-induced menopause as they can be toxic to the ovaries. (5) Short of menopause, these women may also develop premature ovarian insufficiency from depletion of their ovarian reserves and reduction in oocyte quality from DNA damage. On average, there is a 20% risk of permanent chemotherapy-induced ovarian failure following current breast cancer treatment regimen in women below 40 years. (8)(6)

Infertility associated with chemotherapy has negative impact on the patienťs quality of life and moreso patient outcomes as it has been found to influence adherence to systemic chemotherapy in over a quarter of cases. (11)

Fertility preservation refers to the procedure of freezing one’s eggs, sperm, embryos or reproductive tissue so that the individual can hopefully have a biological family in the future. The importance of fertility preservation to cancer patients have been demonstrated in previous studies that reported significant improvement in patient satisfaction, and that patients, in attempts to safeguard their fertility, would choose less optimal breast cancer treatment options. (11) Fertility preservation interventions include freezing of oocytes, sperm, embryos, ovarian tissue, and testicular tissue, or the administration of gonadotropin-releasing hormone agonists before and during chemotherapy (12) Of these, cryopreservation in women appears the most suitable, studied, and universally accepted for cancer patients. (13) The American Society of Clinical Oncologists (ASCO) proposes embryo cryopreservation as the safest and most likely successful fertility preservation method. (14)

Despite recommendations that patients are made aware of the possibility of future infertility that comes with the diagnosis of malignancy in the reproductive age group. There is limited data on the depth of discussion between healthcare workers and patients regarding FP following cancer diagnosis. (6) (15) In light of this, the option of Fertility Preservation (FP) should be discussed with the patient early in their management process. (18) A recent study by Fleischer-Djoleto et al among oncologists in Ghana reported that, even though all participating Oncologists were aware that cancer treatment compromises fertility; only 70% counselled their patients on cancer treatment’s impact. Notably, a significant 68% of these cancer specialists commenced cancer treatment without considering fertility preservation, while 32% of them reported discussing the subject if the patient had fertility wishes. (Fleischer-Djoleto et al, 2022). These notwithstanding, scientific data broadly establishing the barriers to uptake of fertility preservation in Ghana and Sub-Saharan Africa remains scanty. This study there sought to explore and ascertain what barriers exist to fertility preservation among these patients with breast cancer undergoing treatment in Ghana.

## MATERIALS AND METHODS

### Study Setting

This is cross-sectional, multicenter, transdisciplinary research (TDR) that used mixed methods. TDR, comprising Gynaecology, general surgery, Radiotherapy & Oncology and Clinical Psychology disciplines, was used to ensure that the study incorporates perspectives from related stakeholders and develop co-ownership of the project and future phases of the study. The study was conducted at the Breast Clinic, Surgical Department of the Korle Bu Teaching Hospital (KBTH) and the National Radiotherapy Oncology and Nuclear Medicine Center, Korle Bu, both in Ghana.

The Breast Clinic at the Surgical Department sees an average of 30–40 new patients a month, majority of whom are below 40 years. The clinic is manned by three Consultant Breast Surgeons, two senior residents and four residents in training, as well as house officers on rotation at the unit and 14 nurses. Majority of the patients seen have advance stage disease due to the lack of early detection of breast cancer in Ghana. Most of these patients tend to require systemic chemotherapy as part of their treatment. This is done either prior to surgery or after surgery.

The National Radiotherapy Oncology and Nuclear Medicine Centre attends to approximately 70 cases daily, of whom breast cancer constitutes the majority. This center receives referrals nationwide and from other African countries. About 65% of these cases present with advance stages of breast cancer. The center is manned by Consultant and Specialist Radio-oncologists, Medical Officers, Nurses, Medical Physicists and Radiation Therapists. The center runs weekly breast clinics that attend to about 16–20 cases of new breast cancer weekly.

### Study Population

The study population comprised women diagnosed with breast cancer who are under 40 years and being worked up to start or have started treatment at the two centers. Those severely ill, completed childbearing, do not intend having biological children or those using contraception were excluded.

### Data Collection

Power analysis was performed using the prevalence on knowledge on fertility preservation (32%) (27) among cancer patients, the desired level of confidence (95%) and acceptable margin of error (5%) to determine the minimum sample size required to detect a statistically significant effect in the study.

Data was obtained using structured, pretested questionnaires administered by trained research assistants to participants after informed consent had been obtained. These were administered to every consecutive eligible patient at the two study sites on each clinic day over the study period. Data was obtained on patients‘ sociodemographic characterisitics (age, educational level, religion, marital status, parity, occupation, awarness etc), and other key variables including: duration of breast cancer diagnosis, type of treatment(s) received, level of specialist care, whether fertility preservation counselling had been done or not, forms of fertility preservation discussed, patient’s acceptance or otherwise, reasons for not accepting, patient’s perceived barriers, etc.

#### Data Analysis:

Resulting data were entered into Microsoft Excel spreadsheet, cleaned and exported into SPSS version 22 for analysis. Socio-demographic characteristics were analyzed with descriptive statistics, frequencies, proportions and ratios as well as mean ± standard deviation; and results presented in text, figures, frequency tables, charts and graphs.

Test of associations were conducted using the group student t- test for continuous variables and Chi-Square used for categorical data set and the 95% level of significance determined. Logistic regression was used to determine significant association between the participants' sociodemographic characteristics, medical parameters; and their knowledge and perspectives on fertility preservation using odds ratios and confidence intervals (at 95% significance level). P-value below 0.05 was considered statistically significant.

## RESULTS

The study included 300 breast cancer patients with a mean age of 44.47 years (SD ± 1.21). Participants were predominantly aged 41–45 years (38%) and 46–49 years (20%), with fewer participants (0.7%) aged 20–25 years. Most participants were married (60.3%), with 20% being single, 8% divorced, and 5.3% widowed. Educational levels varied considerably, with 29.3% having junior high school (JHS)/ordinary level education and 26.3% attaining tertiary education. Urban residents constituted the majority (84.6%), while rural dwellers made up only 15.3% of the cohort. Importantly, 81.3% of participants had no living children, highlighting the relevance of fertility preservation for a significant portion of the group (Table 1).

Regarding participants' awareness of breast cancer symptoms, lump symptoms were the most reported across all marital groups, particularly among married participants (57.7%), followed by single (22.8%) and those cohabiting (5.7%) (Table 2). Pain in the breast and nipple discharge were other less frequently reported significant initial symptoms.

Awareness of fertility preservation procedures and options was limited (39.3%) among the 41.7% of participants who were below 40years old or less, with hospitals and the internet were the primary sources of information, with 39.2% and 64.0% of informed participants, respectively, indicating they acquired knowledge through these channels (p < 0.023, Table 4). Awareness derived from social media, newspapers, or magazines was notably lower. Regarding familiarity with specific fertility preservation techniques, only 49.1% were aware of oocyte freezing, while 75% knew about embryo freezing among those who reported knowledge (p < 0.029). The vast majority of respondents understand fertility preservation to be about the ability to give birth in the future.

**Figure 1:**
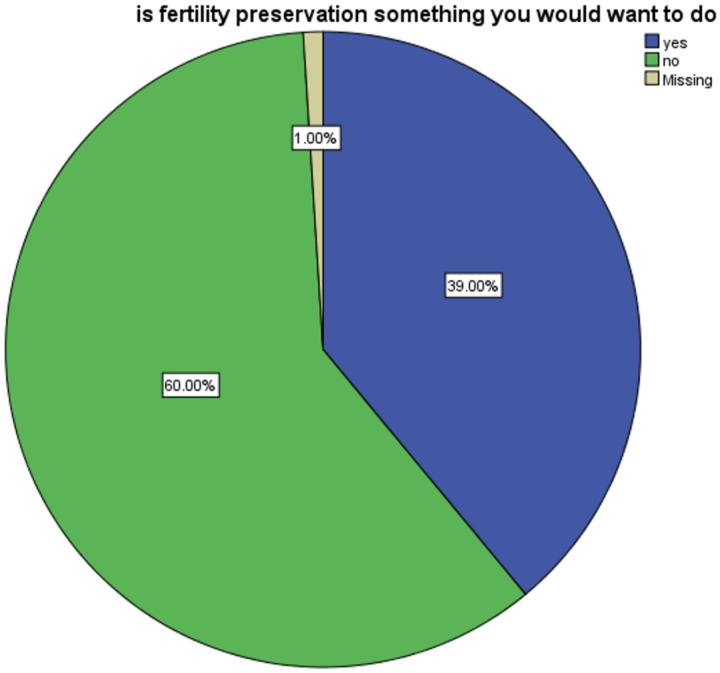
Participants’ attitude toward fertility preservation uptake

Regarding participants’ attitudes toward fertility preservation, 39% indicated they would want to pursue the intervention. (Fig. 1). Several barriers to fertility preservation were identified, with cost emerging as the most significant. Among those who preferred fertility preservation, 73.5% cited cost as a major barrier compared to only 3.4% who did not identify cost as an obstacle (p < 0.00) (Table 3). Similarly, fear and partner objection were significant barriers reported by 50% and 14.3%, respectively, of participants who expressed interest in fertility preservation (p < 0.036 and p < 0.020). Lack of interest was also a prominent factor. Additionally, 83.1% of participants who deemed fertility preservation “not important” opted out of pursuing it (p < 0.00).

Age, parity, marital status, and the desire for children strongly influenced preferences for fertility preservation. Younger participants aged 31–35 years exhibited the highest preference (79.5%) for fertility preservation compared to 30% among those aged 46–50 years (p < 0.000) (Table 5). Parity played a significant role; participants without children showed the strongest preference for fertility preservation (89.3%) compared to those with children (p < 0.007). Marital status also shaped preferences: single (79.7%) and cohabiting (84.2%) participants were more inclined toward fertility preservation than their married (37%) and widowed (6.2%) counterparts (p < 0.001). Furthermore, a strong desire to have children post-treatment was a key determinant, with 87.3% of participants wishing for children expressing interest in fertility preservation (p < 0.001) (Table 5). A substantial proportion (60%) of participants expressed an unwillingness to pursue fertility preservation. This reluctance was predominantly linked to barriers such as cost, fear, and lack of information. Notably, 60% of participants expressed a strong likelihood of advocating for fertility preservation, highlighting its perceived importance when adequately understood (Table 6). This reflects a need for targeted interventions to address knowledge gaps, financial constraints, and social factors influencing fertility preservation decisions.

## DISCUSSION

This study provides critical insights into the barriers to fertility preservation (FP) among breast cancer patients in Ghana, highlighting the interplay of economic, cultural, social, and knowledge-based challenges. While similar barriers have been documented globally, this study offers a nuanced understanding of how these barriers manifest in the Ghanaian context, where healthcare infrastructure, socio-economic disparities, and cultural norms play a pivotal role in shaping patient choices.

The significant influence of cost on fertility preservation decisions underscores the economic challenges faced by cancer patients in LMICs. In this study, 73.5% of participants who preferred fertility preservation identified cost as a barrier. This finding is consistent with studies from other low-resource settings, such as Kenya and Nigeria, where high out-of-pocket expenses limit access to cancer treatments and adjunctive fertility services (14)(15). Fertility preservation services, including oocyte and embryo freezing, require advanced technology, specialized expertise, and long-term storage, which increase costs disproportionately in resource-limited settings (16). Similarly, others have reported that patients do not opt for fertility preservation due to the associated costs, concerns about delaying cancer treatment, non-referral from medical professionals, and their relationship status (17)(18)(19).

The lack of comprehensive health insurance schemes further exacerbates these challenges, emphasizing the urgent need for subsidized FP services or national programs that integrate FP into oncology care financing frameworks.

Cultural norms and social dynamics significantly influenced decisions regarding fertility preservation in this study. Partner objection, reported by 14.3% of participants, emerged as a noteworthy barrier, reflecting the gendered power dynamics in reproductive decision-making within traditional African societies. The factor of the patient’s partner’s support has been reported in previous studies and linked to the patient’s relationship status (19)(21). Fear was another critical factor. Fear may stem from misconceptions about fertility preservation procedures, concerns about delaying cancer treatment, or anxiety about cancer recurrence, which are common themes among patients in LMICs (22).

Additionally, 90.5% of participants who expressed “no interest” in FP as a barrier tied this to fertility considerations being less important than cancer survival. Cultural stigmas around infertility may further complicate discussions about FP, as women diagnosed with cancer may prioritize survival over reproductive health due to societal pressures. These findings highlight the need for culturally tailored interventions to address misconceptions and provide support for informed decision-making.

Limited knowledge and awareness of FP options were evident, with hospitals and the internet emerging as primary sources of information. Despite this, awareness of specific procedures like oocyte freezing (49.1%) and embryo freezing (75%) was low. This may be due to absence of discussions around fertility preservation routinely as part of breast cancer care. Consistently, previous studies in LMICs have shown that many cancer patients are unaware of their fertility risks or preservation options due to inadequate counseling or a lack of trained healthcare professionals to initiate these discussions (20)(12). This is despite recommendations by the American Society of Reproductive Medicine that as part of education and informed consent before cancer therapy, oncologists should address the possibility of infertility with patients in their reproductive years, discuss possible fertility preservation options or refer patients to reproductive specialists (11).

Interestingly, the internet was a significant source of information for participants in this study (64.0%, p < 0.023). This finding aligns with global trends, indicating that patients increasingly turn to online platforms for health information. However, reliance on online sources in LMICs raises concerns about misinformation and unequal access, particularly for rural and less-educated populations (23). In this study, most participants were urban dwellers and had junior high education. Expanding hospital-based education and integrating FP counseling into cancer care pathways is essential for bridging these knowledge gaps.

The desire for FP was strongly associated with younger age and lower parity. Participants aged 31–35 years exhibited the highest preference (79.5%), while interest declined sharply among women aged 46–49 years (30%). These findings reflect global trends, as younger women with fewer children are more likely to view FP as a priority (24).

Marital status also influenced preferences, with single (79.7%) and cohabiting (84.2%) women demonstrating greater interest in FP compared to married or widowed participants. This may be partially due to the married women already having had children or completed their families. Previous studies have consistently reported that single women may perceive greater potential for future childbearing, while older, widowed women may deprioritize FP due to age-related fertility concerns or diminished reproductive aspirations (16).

Additionally, 87.3% of participants who expressed a desire for children after treatment showed interest in FP, underscoring the centrality of future fertility in shaping the preferences of breast cancer patients. This is consistent with findings from North American and European studies, where future family-building goals are critical drivers of FP decisions (25).

This reluctance of 60% of participants to undertake fertility preservation underscores the complex interplay of barriers, including financial constraints, fear, and social stigma. Lack of routine professional discussion of the subject during the treatment planning could be a factor. An earlier study in Ghana found that, although all related medical specialists were aware of fertility preservation, only 32% referred younger cancer patients with early-stage disease and fertility wishes to reproductive specialists for FP (13). Studies in similar settings have noted that the lack of integrated fertility counseling in oncology care can lead to missed opportunities for FP uptake (22)(23). It is instructive that 60% of participants were very likely to advocate for FP. This reflects a need for targeted interventions to address knowledge gaps, financial constraints, and social factors influencing fertility preservation decisions. Further, this observation highlights a latent potential uptake for this intervention if breast cancer patients are appropriately counselled and the financial, educational, and cultural barriers are addressed.

## CONCLUSION

These findings underscore the need for improved awareness, reduced costs, and integration of fertility preservation discussions into breast cancer care in Ghana.

Significant barriers to fertility preservation among breast cancer patients in Ghana include financial constraints, limited knowledge, fear, and partner objection. Younger age, lower parity, and a strong desire for future childbearing were key determinants of interest in fertility preservation. However, overall uptake remains low due to systemic, financial, low awareness, social, and cultural barriers. Addressing these barriers through subsidized fertility preservation services, public awareness campaigns, and culturally tailored, multidisciplinary fertility counseling is essential for improving access and uptake. Integrating discussion around fertility preservation into routine oncology care in Ghana could enhance reproductive autonomy and quality of life for breast cancer patients and survivors.

### Recommendations for Policy and Practice

This study highlights actionable opportunities to improve fertility preservation uptake in Ghana. Based on these findings, the following are recommended for policymakers, clinicians and healthcare providers:

- Subsidizing and integrating fertility preservation services into cancer care packages and expanding health insurance coverage for fertility preservation procedures would address cost-related barriers.
- Capacity building for healthcare professionals to provide culturally sensitive, partner-inclusive family planning counseling is essential for addressing social and gender-related barriers.
- Leveraging accessible media platforms, including radio, television, social media and community outreaches, can improve awareness of fertility preservation among the general and underserved populations for improved uptake.
- Establishing Multidisciplinary Care Models delivered by teams comprising oncologists, reproductive specialists, and counselors can ensure timely and comprehensive fertility preservation discussions.

### Strengths and Limitations

To the best of our knowledge, this study has been the first to assess the important subject of fertility preservation in the West African Sub-region with instructive findings for potential policy that would ultimately improve the outlook and quality of life for breast cancer patients in Ghana and beyond.

The study’s sample size of 300 also strengthens the power of its findings and recommendations which can be generalizable for policy, and clinical interventions.

This study’s findings may be limited by the limitations inherent in the use of cross-sectional design. Additionally relying on participants recall and perspectives to obtain this primary data on a potentially sensitive subject may introduce some bias. The location of this study in two urban centers in one region constitute some geographic restriction that may not make findings truly representative of the entire Ghana.

Future research should adopt longitudinal approaches that examine changes in levels of awareness and attitudes towards fertility preservation with a focus including rural and socio-economically disadvantaged populations. Investigating the impact of targeted interventions, such as financial subsidies, health insurance coverage and enhanced comprehensive counseling programmes, would further address financial and access barriers while also providing valuable insights for scaling up and improving fertility preservation uptake in LMICs for improved quality of life for breast cancer patients.

## Figures and Tables

**Table 1:** Baseline demographic profile of the study subjects

Characteristics		N (%)
**Age:**	20–25	2(0.7)
	26–30	24(8.0)
	31–35	39(13.0)
	36–40	60(20.0)
	41–45	115(38.0)
	46–49	60(20.0)
**Marital status:**	Single	60(20.0)
	Cohabiting	19(6.3)
	Married	181(60.3)
	Divorced	24(8.0)
	Widowed	16(5.3)
**Educational level:**	No formal education	19(6.3)
	Basic education	24(8.0)
	JHS/Ordinary level	88(29.3)
	SHS/Advanced level	76(25.3)
	Tertiary education	79(26.3)
	Post graduate studies	14(4.7)
**Occupational level:**	Accountant	11(3.7)
	Administrator	2(0.7)
	Bankers	4(1.3)
	Artisans	60(20.0)
	Entrepreneur/traders	111(37.0)
	Healthcare professional	12(4.0)
	Researcher	2(0.7)
	Security personnel	8(2.7)
	Pharmacist	1(0.3)
	Teacher	12(4.0)
	Unemployed	43(14.3)
	Cannot disclose my occupation	3(1.0)
	Other	31(10.3)
**Address:**	Rural	46(15.3)
	Urban	254(84.6)
**Parity:**	Living children	56(18.7)
	No children	244(81.3)
**Total**		300(100)

**Table 2:** Symptoms Experienced Leading to Breast Cancer Diagnosis Across Marital Status

Symptoms	Single (%)	Cohabiting (%)	Married (%)	Divorced (%)	Widowed (%)
**Lump symptoms**	56 (22.8)	14 (5.7)	142 (57.7)	21 (8.5)	3 (5.3)
**Pain in breast**	13 (20.6)	6 (9.5)	36 (57.1)	7 (11.1)	1 (1.6)
**Nipple discharge**	7 (21.2)	1 (3.0)	19 (57.6)	6 (18.2)	0 (0.0)
**Breast skin changes**	2 (33.3)	1 (16.7)	1 (16.7)	1 (16.7)	1 (16.7)
**Ulcers/sore**	3 (30.0)	1 (10.0)	4 (40.0)	1 (10.0)	1 (10.0)
**Changes in breast size**	1 (7.1)	0 (0.0)	8 (57.1)	1 (7.1)	4 (28.6)

**Table 3:** Association between fertility concerns and barriers to fertility preservation.

Barrier	Prefer to do fertility preservation – Yes	No	Unsure	p-value
**Cost**	111 (73.5%)	15 (9.9%)	25 (16.6%)	< 0.001
	5 (3.4%)	113 (77.9%)	27 (18.6%)	
**Religious reasons**	0 (0.0%)	1 (50.0%)	1 (50.0%)	0.370
	111 (39.5%)	127 (43.2%)	51 (17.3%)	
**Partner objection**	1 (14.3%)	2 (28.6%)	4 (57.1%)	0.020
	115 (99.1%)	126 (98.4%)	48 (16.6%)	
**Fear**	3 (50.0%)	1 (16.7%)	2 (33.3%)	0.036
	113 (39.0%)	127 (43.8%)	50 (17.2%)	
**Not interested**	1 (1.6%)	57 (90.5%)	5 (7.9%)	< 0.001
	114 (49.4%)	71 (30.7%)	46 (19.9%)	
**Do not believe it is possible**	5 (23.8%)	8 (38.1%)	8 (38.1%)	0.080
	110 (40.1%)	120 (43.8%)	44 (16.1%)	
**Not important**	0 (0.0%)	54 (83.1%)	11 (16.9%)	< 0.001
	116 (50.2%)	74 (32.0%)	41 (17.7%)	
**Effect of cancer disease**	0 (0.0%)	4 (100.0%)	0 (0.0%)	0.070
	116 (39.9%)	124 (42.6%)	51 (17.5%)	

**Table 4:** Awareness and Familiarity with Fertility Preservation among Breast Cancer Patients (< 40 years)

Source of Information on Fertility Preservation	Yes	No	p-value
**Radio**	18 (15.7%)	97 (84.3%)	0.457
	0 (0.0%)	3 (100%)	
**Television**	23 (20.0%)	91 (79.1%)	0.675
	0 (0.0%)	3 (100%)	
**Newspaper/Magazine**	3 (2.6%)	112 (97.4%)	0.777
	0 (0.0%)	3 (100%)	
**Internet**	22 (19.1%)	93 (80.9%)	**0.001**
	3 (100%)	0 (0.0%)	
**Hospital**	51 (44.3%)	64 (55.7%)	0.126
	0 (0.0%)	3 (100%)	
**Family**	6 (5.2%)	109 (94.8%)	0.685
	0 (0.0%)	3 (100%)	
**Friend**	18 (15.7%)	97 (84.3%)	0.457
	0 (0.0%)	3 (100%)	
**Social media**	18 (15.7%)	97 (84.3%)	0.457
	0 (0.0%)	3 (100%)	
Familiarity with Types of Fertility Preservation Procedures (Among Those Aware and Responded)			
Procedure	Yes	No	
Hormonal injection	15 (31.9%)	32 (68.1%)	
Oocyte (egg) freezing	28 (49.1%)	29 (50.9%)	
Embryo freezing	6 (75.0%)	2 (25.0%)	
Not sure	4 (80.0%)	1 (20.0%)	**0.029**

Values are presented as n (%). p-values from chi-square or Fisher’s exact test.

**Table 5:** Association Between Fertility Concerns and Variables of Interest

	Category	Yes (%)	No (%)	Unsure (%)	p-value
**Age**	20–25	2 (100.0)	0 (0)	0 (0)	< 0.001
	26–30	17 (70.8)	4 (16.7)	3 (12.5)	
	31–35	31 (79.5)	6 (15.4)	2 (5.1)	
	36–40	27 (45.0)	30 (50.0)	3 (5.0)	
	41–45	44 (38.6)	66 (57.9)	4 (3.5)	
	46–49	18 (30.0)	38 (63.3)	4 (6.7)	
**Parity**	Para 0	50 (89.3)	5 (8.9)	1 (1.8)	0.007
	Para 1	31 (63.3)	16 (32.7)	2 (4.1)	
	Para 2	37 (45.7)	37 (45.7)	7 (8.6)	
	Para 3	11 (17.2)	48 (75.0)	5 (7.8)	
	Para 4	9 (25.7)	25 (71.4)	1 (2.9)	
	Para 5	1 (9.1)	10 (90.9)	0 (0.0)	
	Para 6	0 (0.0)	3 (100.0)	0 (0.0)	
**Marital Status**	Single	47 (79.7)	11 (18.6)	1 (1.7)	< 0.001
	Cohabiting	16 (84.2)	2 (15.8)	0 (0.0)	
	Married	67 (37.0)	102 (56.4)	12 (6.6)	
	Divorced	8 (33.3)	13 (54.2)	3 (12.5)	
	Widowed	1 (6.2)	15 (93.8)	0 (0.0)	
**Educational Level**	No Formal Education	6 (31.6)	13 (68.4)	0 (0.0)	0.438
	Basic Education	13 (54.2)	11 (45.6)	0 (0.0)	
	JHS/Ordinary Level	36 (40.9)	44 (50.0)	8 (9.1)	
	SHS/Advanced Level	40 (52.6)	33 (43.4)	3 (3.9)	
	Tertiary Education	5 (38.5)	7 (53.8)	1 (7.7)	
**Treatment Modality**	Surgery	64 (43.8)	78 (53.4)	4 (2.7)	0.034
		38 (41.3)	44 (47.8)	10 (10.9)	
	Chemotherapy	92 (44.0)	106 (50.7)	11 (5.3)	0.686
		89 (40.5)	119 (54.1)	12 (5.5)	
	Radiotherapy	34 (43.6)	40 (51.3)	4 (5.1)	0.982
		68 (42.8)	82 (51.6)	9 (5.7)	
	Palliation	13 (76.5)	3 (17.6)	1 (5.9)	0.046
		89 (40.5)	119 (54.1)	12 (5.5)	
**Reproductive Intentions**	Wish to have children after treatment	124 (87.3)	17 (12.0)	1 (0.7)	< 0.001
		8 (6.1)	122 (92.4)	2 (1.5)	
	Considering having children after treatment	139 (100.0)	0 (0.0)	0 (0.0)	
		0 (0.0)	144 (100.0)	0 (0.0)	
		0 (0.0)	0 (0.0)	16 (100.0)	

Values are presented as n (%).

**Table 6:** Fertility likelihood

Likelihood of telling other about fertility preservation	frequency	percentage (%)
Very likely	181	60
Likely	90	30
Neutral	14	4.7
Unlikely	11	3.7

## Data Availability

The data supporting the findings of this study are available from the corresponding author upon reasonable request.
